# Sustained remission with methotrexate monotherapy after 22-week induction treatment with TNF-alpha inhibitor and methotrexate in early psoriatic arthritis: an open-label extension of a randomized placebo-controlled trial

**DOI:** 10.1186/s13075-019-1998-4

**Published:** 2019-09-14

**Authors:** Henriëtte M. Y. de Jong, Leonieke J. J. van Mens, Michael T. Nurmohamed, Marc R. Kok, Arno W. R. van Kuijk, Dominique L. P. Baeten, Marleen G. H. van de Sande

**Affiliations:** 1 Department of Clinical Immunology and Rheumatology, Infection & Immunity Institute, Amsterdam UMC, AMC/University of Amsterdam, Meibergdreef 9, 1105 AZ Amsterdam, the Netherlands; 2 Amsterdam Rheumatology & Immunology Center (ARC), Amsterdam, the Netherlands; 3 Department of Rheumatology, Amsterdam UMC, Vrije Universiteit Amsterdam, Amsterdam, the Netherlands; 4 Department of Rheumatology, Reade, Amsterdam, the Netherlands; 5 Department of Rheumatology and Clinical Immunology, Maasstad Hospital, Rotterdam, the Netherlands

**Keywords:** Early psoriatic arthritis, Remission, Treatment withdrawal, Methotrexate, TNF inhibitor

## Abstract

**Background:**

If TNF inhibitors are initiated in the early stages of psoriatic arthritis, this could potentially modulate disease and therefore allow us to discontinue the TNF inhibitor after achieving remission.

**Objective:**

To investigate whether remission induced by tumour necrosis factor alpha inhibitor (TNFi) and methotrexate in patients with early psoriatic arthritis is sustained after withdrawal of TNFi.

**Methods:**

Open-label extension of a recently published double-blind, randomized placebo-controlled trial. Patients with psoriatic arthritis fulfilling the CASPAR criteria and with active disease at baseline (swollen and tender joint count ≥ 3) were randomized to either golimumab and methotrexate or matched placebo and methotrexate. Patients in Disease Activity Score (DAS) remission at week 22 continued in the open-label extension on methotrexate monotherapy. The primary end point was the percentage of patients in DAS-CRP remission (DAS < 1.6) at week 50.

**Results:**

Eight patients from the original placebo group and 18 patients from the original TNFi group continued in the extension phase. At week 50, 6 out of 8 (75%) patients from the original MTX (methotrexate) group versus 10 out of 18 (56%) patients from the original MTX+TNFi group were in DAS-CRP remission (*p* = 0.347). Considering the total study population, 6 out of 24 (25%) of the original MTX group versus 10 out of 26 (38.5%) of the original MTX+TNFi group were in DAS remission at week 50 (*p* = 0.308).

**Conclusions:**

Remission achieved by initial combination treatment with TNFi and methotrexate in early psoriatic arthritis is maintained on methotrexate monotherapy in approximately half of the patients.

**Trial registration:**

Registered at Clinicaltrials.gov with number NCT01871649 on June 7, 2013.

## Introduction

Biologics have drastically changed the field of rheumatology. Whereas they have originally been used to treat patients who were refractory to classical disease-modifying anti-rheumatic drugs (DMARDs) such as methotrexate (MTX), increasing evidence shows that their use in early disease allows to achieve high remission rates [1–4]). Moreover, it has been postulated that if biologics are initiated in the early stages of disease, during the so-called window of opportunity [5], this could promote an immune reset rather than merely suppression of inflammation and thereby alter the course of the disease and allow for discontinuation of treatment in patients who achieve remission.

Most of the studies exploring these concepts have been conducted in rheumatoid arthritis. It remains unclear to what extent these concepts may also apply to psoriatic arthritis (PsA), a subgroup of spondyloarthritis that can present with skin and nail psoriasis, arthritis, enthesitis, dactylitis, and axial disease [6].

We recently demonstrated that initiation of tumor necrosis factor alpha inhibition (TNFi) with golimumab in combination with MTX doubled the number of early PsA patients achieving Disease Activity Score (DAS) remission at week 22 in comparison with MTX monotherapy from 42% with MTX alone to 81% with golimumab plus MTX combination therapy [4]. To explore the hypothesis that remission induced by TNFi plus MTX in patients with early psoriatic arthritis can be sustained after withdrawal of TNFi, we conducted an open-label extension study of this trial with patients that were in DAS-CRP remission at week 22 and continued on MTX monotherapy. Disease activity was assessed at weeks 36 and 50.

## Methods

### Study design and patients

The original study design and baseline characteristics have been described in detail [4]. In short, 51 patients with psoriatic arthritis (PsA), fulfilling the CASPAR criteria and with active disease (defined as swollen and tender joint count ≥ 3), were included. During the double-blind phase (up to week 22), all patients received methotrexate (MTX) up to 25 mg/week and were randomized to either golimumab 50 mg/month (*n* = 26) or matched placebo (*n* = 24). Patients with a status of DAS-CRP remission at week 22, defined by a DAS-CRP score < 1.6, were offered to enter the open-label extension phase up to week 50 on MTX monotherapy. A clinical assessment was done at week 36 and week 50, and an additional visit was done in the case of worsening or recurrence of symptoms. Participants who had loss of remission were withdrawn from the trial. The group that originally used MTX and placebo will further be referred to as the ‘original MTX group’, and the group that used methotrexate and golimumab will further be referred to as the ‘original MTX+TNFi group’.

This study was conducted at three centres in the Netherlands between September 2013 and September 2017 and was approved by the Medical Ethics Committee of the Academic Medical Centre in Amsterdam. Written informed consent was obtained from each patient before enrolment. The study was conducted in compliance with the International Conference on Harmonisation Good Clinical Practice guidelines and the Declaration of Helsinki and is registered at clinicaltrials.gov under NCT01871649.

### Assessments

The primary efficacy end point of this study was the percentage of patients who sustained a status of DAS-CRP remission [7] (DAS-CRP score < 1.6) at week 50. Secondary efficacy end points included Low Disease Activity (LDA) status (DAS-CRP < 2.4), criteria for Minimal Disease Activity (MDA) [8], and Disease Activity in Psoriatic Arthritis Low Disease Activity (DAPSA-LDA) [9]. Clinical evaluations, patient-reported outcomes, and standard laboratory tests were done at every study visit. Safety end points included adverse events (AEs) and serious AEs (SAEs) and discontinuation or interruption of study treatment.

### Statistical analysis

Data are presented as mean (SD) unless indicated otherwise. Differences between both groups were analysed with a chi-square test for categorical data and a Mann-Whitney *U* test for continuous data. The primary and secondary outcomes were analysed with an intention-to-treat analysis. Patients that discontinued for any reason during the extension study were considered non-responders, as were patients that were in remission at week 22 but did not attend a study visit during the extension study. All statistical tests were two sided, and a *p* value of < 0.05 was considered statistically significant.

## Results

### Study population and patient disposition

**Fig. 1:**
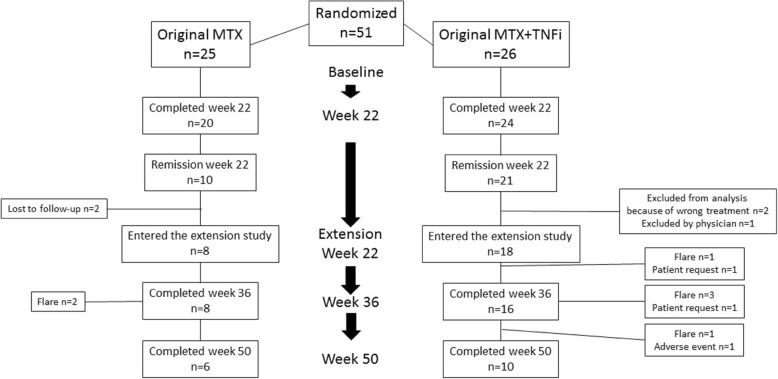
The patient disposition and flow chart

The patient disposition and flow chart are summarized in Fig. 1. Ten patients from the original MTX group achieved remission in the first 22 weeks of the study. Of those, 2 were lost to follow-up. Therefore, 8 patients from the original MTX group entered the extension phase, of whom 6 completed week 50. For the original MTX+TNFi group, 21 patients achieved remission in the first 22 weeks of the study. Three were excluded before the start of the extension phase; therefore, 18 patients entered the extension phase, of whom 10 completed week 50.

Baseline characteristics of the total study cohort have been described previously [4]. Table 1 shows demographics, disease characteristics, and disease activity measures of the 26 patients (18 from the original MTX+TNFi group and 8 from the original MTX group) continuing in the extension phase for baseline and weeks 22, 36, and 50 (data as observed). Demographics did not differ between both groups. At baseline VAS, patient pain was lower in the original MTX group compared to that in the original MTX+TNFi group (*p* = 0.011), as was the BASDAI (*p* = 0.047). At week 22, PASI score was significantly higher in the original MTX group (*p* = 0.001). At weeks 36 and 50, there were no differences between both groups. The overall mean (SD) dosage of methotrexate in the extension phase was comparable in both groups: 22.5 (4.9) mg/week in the original MTX group and 20.9 (6.2) mg/week in the original MTX+TNFi group (non-significant).

**Table 1:** Demographics, characteristics, and disease activity measures as observed on patients who entered the extension study

	Baseline			Week 22			Week 36			Week 50		
	Original MTX (*n* = 8)	Original MTX+TNFi (*n* = 18)	*p*	Original MTX (*n* = 8)	Original MTX+TNFi (*n* = 18)	*p*	Original MTX (*n* = 8)	Original MTX+TNFi (*n* = 16)	*p*	Original MTX (*n* = 6)	Original MTX+TNFi (*n* = 10)	*p*
Age	46 (13.1)	48.7 (10)	NS									
Gender (male/female)	7/1	13/5	NS	7/1	13/5	NS	7/1	13/3	NS	5/1	9/1	NS
Disease duration < 2 years, *n* (%)	5 (62.5)	14 (77.8)	NS	5 (62.5)	14 (77.8)	NS	5 (62.5)	12 (75)	NS	4 (66.7)	6 (60)	NS
SJC 66, median (IQR)	3.5 (3–7.3)	7 (4–8.3)	NS	0.5 (0–2)	0 (0–1)	NS	1 (0.3–2)	0 (0–1.3)	NS	0 (0–1.3)	0 (0–3.3)	NS
TJC 68, median (IQR)	5 (4–13.8)	10 (4.8–14.3)	NS	1 (0.3–3.3)	0 (0–3.3)	NS	1.5 (0.3–2.8)	1.5 (0–4.5)	NS	0 (0–1.5)	1.5 (0–4.3)	NS
PASI score, median (IQR)	2.7 (0.5–6)	1.9 (0.8–3.8)	NS	0.8 (0.4–1.7)	0	0.001	0.5 (0.5–1.8)	0	NS	1 (0.3–1.9)	0.6 (0–1.3)	NS
PASI > 2.5, *n* (%)	4 (50)	8 (44.4)	NS	1 (12.5)	0	NS	0	1 (6.3)	NS	0	1 (10)	NS
Pts with enthesitis, *n* (%)	0	2 (11.1)	NS	1 (12.5)	1 (5.6)	NS	1 (12.5)	3 (18.9)	NS	0	1 (10)	NS
Pts with dactylitis, *n* (%)	4 (50)	4 (22.2)	NS	1 (12.5)	0	NS	4 (50)	1 (6.3)	NS	1 (16.7)	1 (10)	NS
ESR (mm/h), median (IQR)	15.5 (5.5–24.3)	20.5 (4.3–31.8)	NS	7 (3.5–15.8)	2 (2–18)	NS	5.5 (2–10.8)	5 (2–6.5)	NS	5 (2.5–11.5)	6 (2–12)	NS
CRP (mg/L), median (IQR)	9.5 (2.1–14)	4 (1.3–14.5)	NS	3.1 (0.9–15)	1.1 (0.4–2.9)	NS	1.8 (0.7–8)	1.5 (0.4–4)	NS	4.2 (0.9–7.4)	2 (1–5)	NS
VAS patient global (mm)	26.9 (17.8)	46.3 (24.9)	NS	23.4 (25.9)	17.7 (17.6)	NS	18.3 (20.9)	24.1 (23.8)	NS	17.4 (15.2)	19.8 (20)	NS
VAS patient pain (mm)	20 (15.8)	47.7 (26.1)	0.011	8.1 (12.7)	13.6 (17.5)	NS	9.4 (8.7)	23 (21.6)	NS	6.2 (4)	21 (27.1)	NS
VAS physician (mm)	43.1 (14.0)	45.3 (15.6)	NS	12.6 (10.2)	8.1 (9.3)	NS	8.9 (8.1)	13.8 (19.6)	NS	15.6 (12.1)	13 (17.5)	NS
BASDAI	2.4 (1.8)	4.1 (1.9)	0.047	1.5 (1.6)	1.8 (1.5)	NS	1.7 (1.5)	2 (1.7)	NS	1.8 (1.7)	1.7 (1.4)	NS

### Safety

During the extension phase, one serious adverse event (SAE) occurred in a patient from the original MTX+TNFi group: a small bowel obstruction with surgical intervention, which was judged unrelated to the study and did not lead to early termination. Eighteen AEs occurred during the extension phase: 9 in the original MTX+TNFi group and 9 in the original MTX group. One patient from the original MTX+TNFi group discontinued after week 40 because of an AE related to the study medication (liver enzymes > 2 times the upper limit of normal).

### Efficacy

Six out of 8 (75%) patients from the original MTX group completed the extension study and maintained DAS remission at week 50. Five out of 6 fulfilled criteria for MDA, and all were in DAPSA-LDA. Two patients had a loss of DAS remission at week 36; 1 had a status of LDA according to the DAS, 1 was in DAPSA-LDA, and none were in MDA.

Ten out of 18 patients (56%) from the original MTX+TNFi group completed the extension study and maintained DAS remission at week 50. Six out of 10 fulfilled the criteria for MDA, and 7 out of 10 were in DAPSA-LDA. Of the 8 patients dropping out of this arm of the study, 3 patients discontinued while still in DAS remission but were considered non-responders in the intention-to-treat analysis: 1 discontinued because of an AE (week 40) and 2 discontinued upon their own request (weeks 29 and 36) (Fig. 1). Five patients had a loss of DAS remission (1 at week 29, 3 at week 36, and 1 at week 45); 4/5 patients had a status of LDA according to the DAS, 1/5 was in MDA, and 3/5 in DAPSA-LDA.

In the intention-to-treat analysis, 6/8 (75%) patients from the original MTX group versus 10/18 (56%) patients from the original MTX+TNFi group were in DAS-CRP remission at week 50 (*p* = 0.347). Considering not only the extension phase but the complete study from baseline to week 50, 6/24 (25%) patients from the original MTX group had a status of DAS-CRP remission at week 50 compared to 10/26 (38%) patients from the original MTX+TNFi group (*p* = 0.308).

## Discussion

We recently reported that initiating combination therapy with MTX+TNFi resulted in doubling the rate of DAS-CRP remission at week 22 (81%) compared to MTX alone in patients (42%) with early psoriatic arthritis (PsA). We hypothesized that achieving remission in this ‘window of opportunity’ would allow to maintain clinical benefit in a substantial number of patients even after stopping the TNFi at week 22. Here we report that 56% of those patients indeed maintained remission up to week 50, whereas 75% of the patient achieving remission at week 22 on MTX monotherapy maintained remission over time. Taking into account the total study population, 38% of the original MTX+TNFi group versus 25% of the original MTX group achieved and maintained remission up to week 50.

A number of important aspects should be taken into consideration when interpreting the data of the present study. First, the number of patients included in the extension phase of the study was small, especially in the original MTX monotherapy group. Second, the study did not assess drug-free remission as all patients were on continuous MTX therapy from baseline to week 50 to reflect standard of care; the study was not designed to assess the real efficacy of MTX. Continuation of MTX in all patients also explains the high maintenance of remission in the original MTX group, as there was no drug withdrawal in this group, only withdrawal of the placebo. Third, the patients and investigators remained blinded during the whole study (up to week 50) for the golimumab versus placebo treatment in the induction phase of the study, but were aware that this initial treatment was withdrawn at week 22. Fourth, we used the most conservative version of the data to do the analyses. Two patients from the original MTX+TNFi group who experienced a flare during the extension phase were actually still in DAS remission, but were nevertheless withdrawn from the study by the study physician because of arthritis in multiple joints not included in the DAS. Although still formally in DAS remission, these patients were considered as having a loss of remission in the analyses. Also patients dropping out for other reasons were considered as non-responders.

Despite these caveats, our findings are concordant with several other studies in PsA and other types of spondyloarthritis [10–12]). Huynh et al. reported that 55.1% of patients with PsA who discontinued TNFi had persistent clinical benefit of TNFi therapy at the last clinical visit. In this study, smoking and higher disease activity at the time of discontinuation were predictors of loss of clinical benefit, but disease duration did not affect the outcome [10].

The data of the current trial fail to support the hypothesis of immune reset by early TNFi treatment in PsA. In summary, our findings indicate that it is possible to maintain remission on MTX monotherapy in a substantial number of patients with early PsA achieving remission by initial combination treatment with TNFi and methotrexate. However, for how long this remission can be maintained and whether the maintained remission in these patients is due to an immune reset or merely due to suppression of inflammation is not known. Moreover, a fair number of patients in the original MTX+TNFi lost remission after stopping TNFi, which shows that our treatment strategy did not provide the ‘window of opportunity’ to change the disease course in all patients. Whether even earlier initiated (combination) treatment or other targeted treatment could provide a ‘window of opportunity’ with sustained remission in all patients needs further assessment in future treatment strategy trials.

## Data Availability

The datasets used and analysed during the current study are available from the corresponding author on reasonable request.

## Abbreviations

TNFi: Tumour necrosis factor inhibitor
PsA: Psoriatic arthritis
MTX: Methotrexate
DMARDs: Disease-modifying anti-rheumatic drugs
DAS: Disease Activity Score
CRP: C-reactive protein
MDA: Minimal Disease Activity
LDA: Low Disease Activity
DAPSA-LDA: Disease Activity in Psoriatic Arthritis Low Disease Activity
AE: Adverse event
SAE: Serious adverse event
VAS: Visual analogue scale
